# Supplementary material for: FlhE functions as a chaperone to prevent formation of periplasmic flagella in Gram-negative bacteria
Source: Nat Commun. 2024 Jul 14;15:5921. doi: 10.1038/s41467-024-50278-0 (PMC11247099; doi:10.1038/s41467-024-50278-0)
Supplement: Supplementary file 1 — Supplementary Information [file 41467_2024_50278_MOESM1_ESM.pdf]

FlhE functions as a chaperone to prevent formation of periplasmic flagella in Gram-negative bacteria

Manuel Halte[1*], Ekaterina P. Andrianova[2], Christian Goosmann[3], Fabienne F.V. Chevance[4], Kelly T. Hughes[4], Igor B. Zhulin[2], Marc Erhardt[1,5*]

[1]Institute of Biology, Humboldt-Universität zu Berlin, Philippstr. 13, 10115 Berlin, Germany

[2]Department of Microbiology, The Ohio State University, Columbus, Ohio 43210, USA

[3]Max Planck Institute for Infection Biology, Charitéplatz 1, 10117 Berlin, Germany

[4]School of Biological Sciences, University of Utah, Salt Lake City, UT, USA

[5]Max Planck Unit for the Science of Pathogens, Charitéplatz 1, 10117 Berlin, Germany

[*]Corresponding author

Supplementary Materials:

Supplementary Figures S1 – S11

Supplementary Materials and Methods

Supplementary Tables S2 – S5

Supplementary References

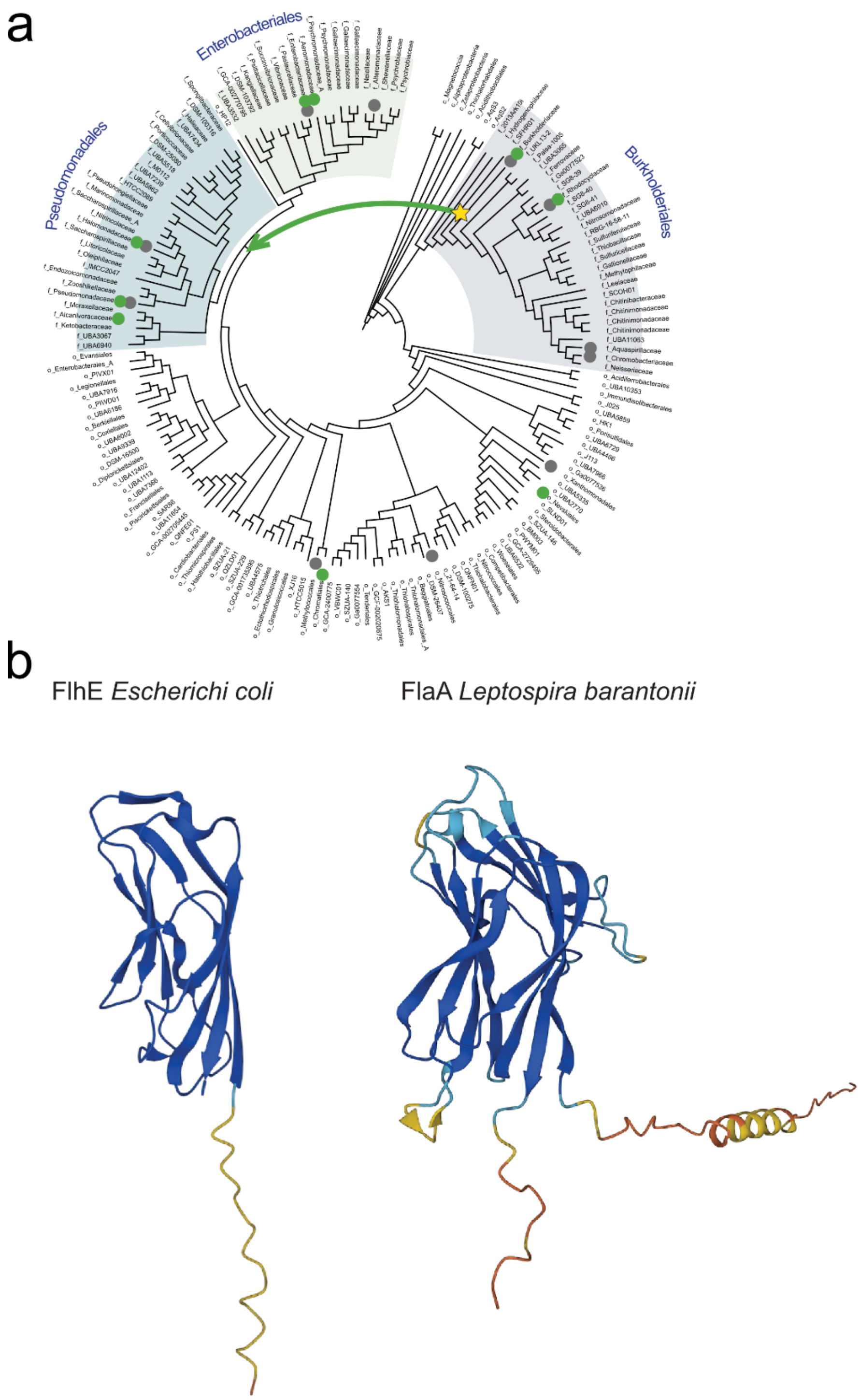

Fig. S1: Distribution and crystal structure of FlhE. **a** Distribution of the FlhE domain across *Gammaproteobacteria*. Green dot indicates the presence of the *flhE* gene in flagellar operons; grey dot indicates the presence of genes coding for FlhE domain containing proteins elsewhere in the genome. Star shows a plausible event of FlhE recruitment into the flagellar apparatus. Green arrow indicates horizontal gene transfer of the flagellar FlhE. **b** The crystal structure of *E. coli* FlhE (PDB: 4QXL) and the AlphaFold model of *Leptospira barantonii* FlaA (A0A5F2BH85_9LEPT) are shown. Both structures adopt the same β-sandwich fold.

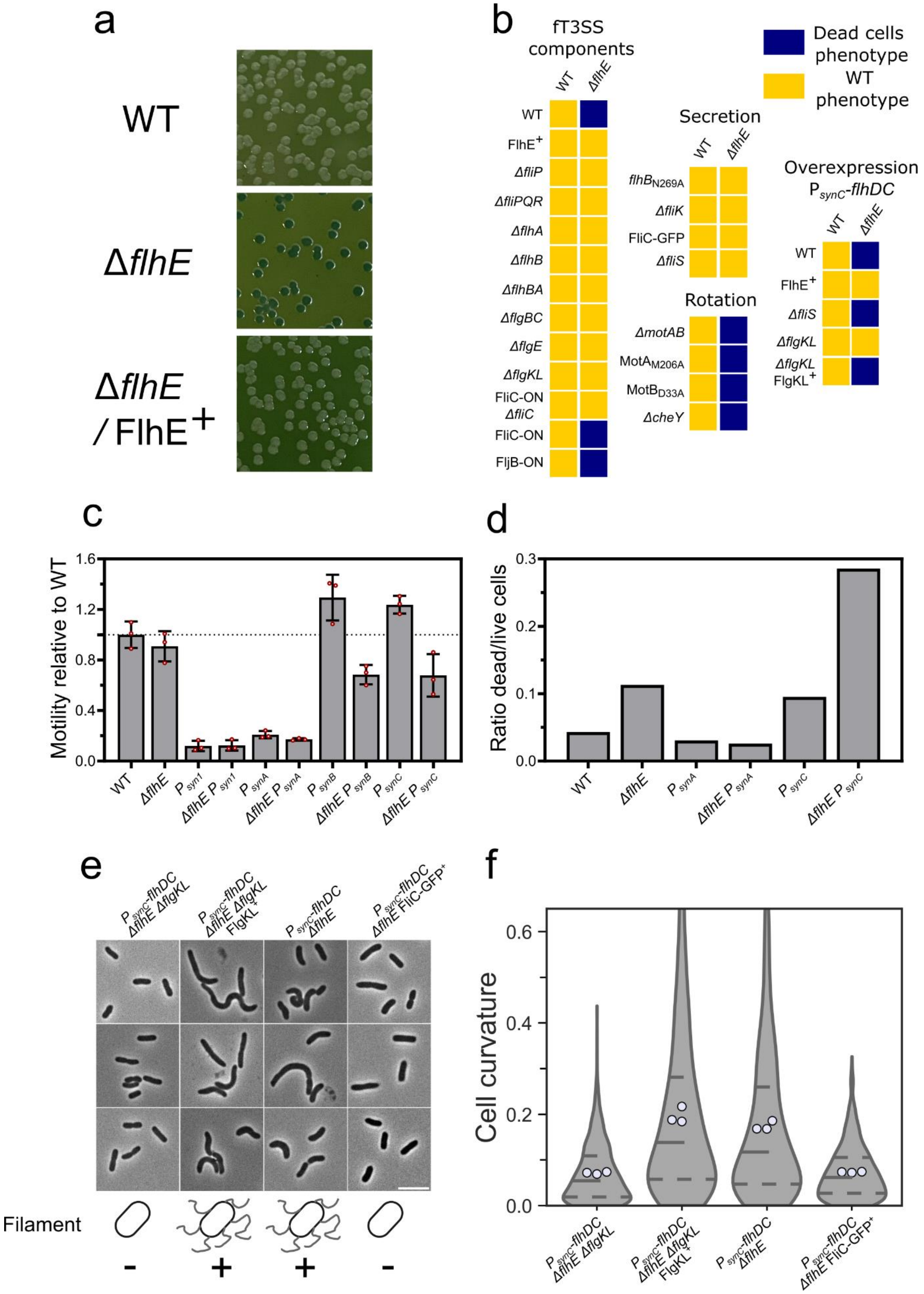
a
WT
ΔflhE
ΔflhE / FlhE+
b
fT3SS components
WT
ΔflhE
WT
FlhE+
ΔfliP
ΔfliPQR
ΔflhA
ΔflhB
ΔflhBA
ΔflgBC
ΔflgE
ΔflgKL
FliC-ON ΔfliC
FliC-ON
FljB-ON
Secretion
flhB N269A
ΔfliK
FliC-GFP
ΔfliS
Rotation
ΔmotAB
MotA M206A
MotB D33A
ΔcheY
Dead cells phenotype
WT phenotype
Overexpression P synC-flhDC
WT
FlhE+
ΔfliS
ΔflgKL
ΔflgKL FlgKL+
c
Motility relative to WT
1.6
1.2
0.8
0.4
0.0
WT
ΔflhE
P syn1
ΔflhE P syn1
P synA
ΔflhE P synA
P synB
ΔflhE P synB
P synC
ΔflhE P synC
d
Ratio dead/live cells
0.3
0.2
0.1
0.0
WT
ΔflhE
P synA
ΔflhE P synA
P synC
ΔflhE P synC
e
P synC-flhDC ΔflhE ΔflgKL
P synC-flhDC ΔflhE ΔflgKL FlgKL+
P synC-flhDC ΔflhE
P synC-flhDC ΔflhE FliC-GFP+
Filament
-
+
+
-
f
Cell curvature
0.6
0.4
0.2
0.0
P synC-flhDC ΔflhE ΔflgKL
P synC-flhDC ΔflhE ΔflgKL FlgKL+
P synC-flhDC ΔflhE
P synC-flhDC ΔflhE FliC-GFP+

Fig. S2: Loss of FlhE causes cell death in the presence of filament formation. **a** Exemplary image of WT and Δ*flhE* mutant streaked on GP. Δ*flhE* mutant formed blue colonies compared to WT and Δ*flhE* $FlhE^+$ expressed constitutively *in trans* from a plasmid formed white colonies. **b** Representation of the phenotype observed on GP after deletion of genes involved in the assembly of fT3SS, secretion and flagella rotation. Yellow square represents WT phenotype and blue square cell lysis. Formation of the flagellar filament in Δ*flhE* background causes cell death. Deletion of basal body components preventing the assembly of the fT3SS restored the WT phenotype. In the presence of hook but absence of filament (FliC-ON Δ*fliC*), WT phenotype can be observed. The same phenotype is observed when blocking the secretion pore with FliC-GFP, indicating secretion itself is not responsible for the Δ*flhE* phenotype. Deletion of stator units (Δ*motAB*) or point mutants preventing the rotation ($MotA_{M206A}$ / $MotB_{D33A}$) did not restore the WT phenotype. **c** Overexpression of FlhDC causes motility decrease in Δ*flhE* background. Motility test in swimming agar were performed at 37°C. Biological triplicates were performed, and motility of the various strains was made relative to WT for each plate. Strong constitutive promoters P_{synB}-*flhDC* and P_{synC}-*flhDC* increase motility in presence of FlhE 1.5-fold relative to WT and is decreased 2-fold in Δ*flhE* background. **d** Propidium staining of WT and Δ*flhE* mutant in native promoter *flhDC*, P_{synA}-*flhDC* and P_{synC}-*flhDC* background. A steep increase in cell death can be observed in P_{synC}-*flhDC* Δ*flhE* background. Data shown are from one representative experiment. **e** Deletion of hook-filament junction (Δ*flgKL*) and expression of FliC-GFP *in trans*, preventing the assembly of the filament and secretion of flagellins restore the WT phenotype in the P_{synC}-*flhDC* Δ*flhE* background. Complementation of Δ*flgKL in trans* causes the shape defect, confirming that the Δ*flgKL* deletion do not cause any polar effect on the *flg* operon. Scale bar = 5 µm. **f** Cell curvature analysis of the cells displayed in **e**. 3 independent biological replicates were performed. Each white dot represents the mean curvature of one biological replicate. The violin plot represents the median and inner quartiles of the whole population. At least 300 cells per replicates were analysed. Source data are provided as a Source Data file.

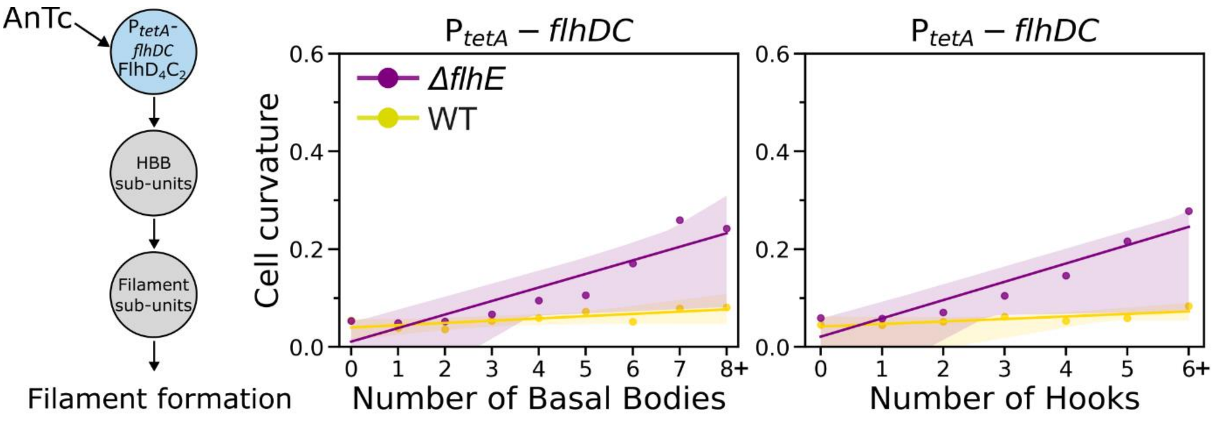

Fig. S3: Cell curvature increase in the Δ*flhE* mutant is correlated with an increase number of basal bodies (BB, FliG-mNeonGreen) and hooks ($FlgE_{S171C}$, Maleimide STAR RED) in an AnTc-inducible P_{tetA}-*flhDC* background. 3 independent biological replicates were performed. At least 250 cells per replicates were analysed using MicrobeJ. Dots represent the cell curvature mean for all cells with a given number of basal bodies or hooks. The curve represents the confidence interval, with an interval CI=99.9 (WT in yellow, Δ*flhE* mutant in magenta). Source data are provided as a Source Data file.

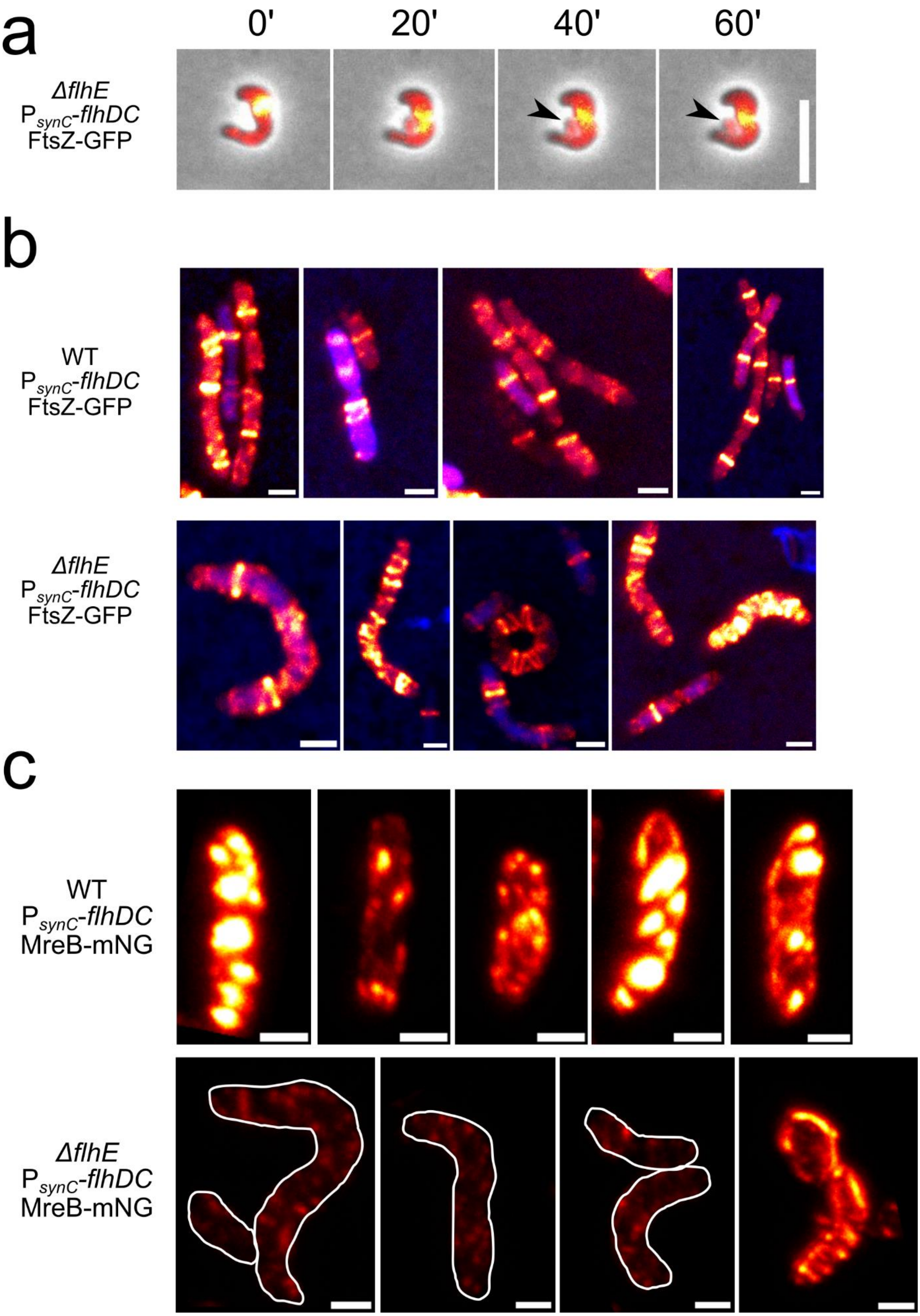

Fig. S4: Representative images of FtsZ-GFP and MreB-mNeonGreen. **a** Time lapse microscopy of plasmid-based expressed FtsZ-GFP in P_{synC}-*flhDC* Δ*flhE* mutant from overnight

culture. OM rupture and release of the cytoplasm occurs at the site of the FtsZ ring position as indicated by the black arrow. Scale bar = 5 µm. **b** Exemplary image of overnight cultures of P_{synC}-*flhDC* and P_{synC}-*flhDC* Δ*flhE* mutant carrying a vector expressing FtsZ-GFP, observed by confocal microscopy. DNA staining was performed with Maleimide LIVE 560 (blue). Scale bar = 1 µm. **c** Exemplary image of overnight cultures of P_{synC}-*flhDC* and P_{synC}-*flhDC* Δ*flhE* mutant carrying a vector expressing MreB-mNeonGreen (G228-D229), observed by confocal microscopy. Data shown are from one representative experiment and have been independently replicated in at least one preliminary experiment. Scale bar = 1µm.

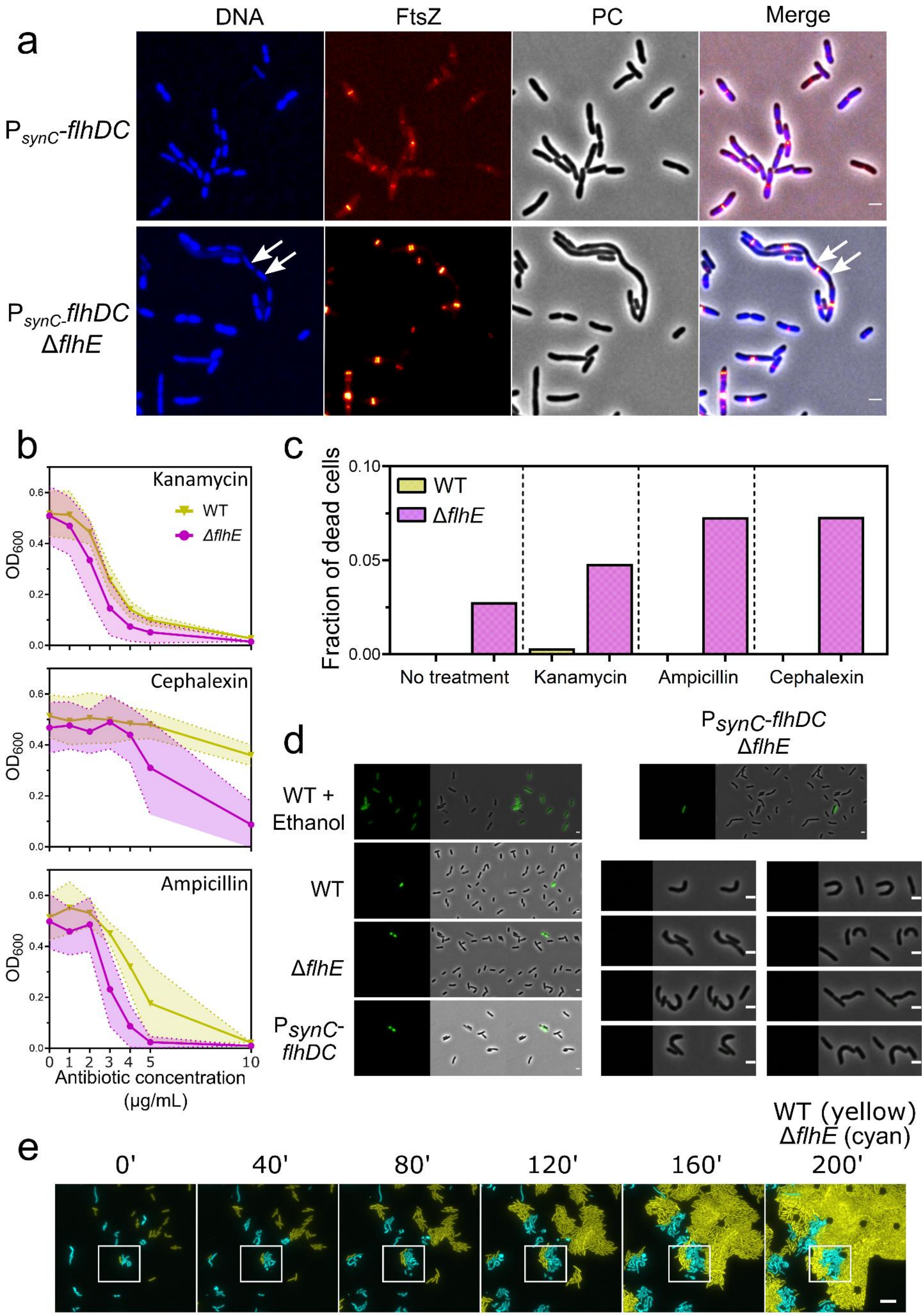

a
DNA
FtsZ
PC
Merge
PsynC-flhDC
PsynC-flhDC ΔflhE
b
Kanamycin
WT
ΔflhE
OD600
Cephalexin
Ampicillin
0 1 2 3 4 5 10
Antibiotic concentration (μg/mL)
c
Fraction of dead cells
0.10
0.05
0.00
WT
ΔflhE
No treatment
Kanamycin
Ampicillin
Cephalexin
d
PsynC-flhDC ΔflhE
WT + Ethanol
WT
ΔflhE
PsynC-flhDC
e
0'
40'
80'
120'
160'
200'
WT (yellow)
ΔflhE (cyan)

Fig. S5: Effect of Δ*flhE* on PG and OM. **a** DNA staining with Maleimide 560 LIVE (blue) of P_{synC}-*flhDC* and P_{synC}-*flhDC* Δ*flhE* expressing FtsZ-eGFP. Scale bar = 2 µm. **b** Killing curve of P_{synC}-*flhDC* and P_{synC}-*flhDC* Δ*flhE* in presence of different antibiotic concentration. Mid-exponential phase cultures were backdiluted to OD_{600} = 0.01 in a transparent clear bottom 96-well plate. After 1 h incubation at 37°C in a plate reader, antibiotic was added at the indicated concentration. Incubation was resumed and OD_{600} after 8 h incubation was plotted for each concentration. Experiment display the mean and standard deviation of 4 biological replicates (WT in yellow, Δ*flhE* mutant in magenta). **c** Impact of antibiotics on cell death in P_{synC}-*flhDC* and P_{synC}-*flhDC* Δ*flhE*. Middle exponential phase cultures were treated with 10 µg/mL of antibiotics for 1.5 h at 37°C and spotted on 1% agarose pad. Cell death was visually assessed in cells losing the contrast indicative of membrane rupture and cytoplasmic contents loss. At least 300 cells per conditions were analysed. Source data are provided as a Source Data file. **d** DNA SYTOX GREEN staining indicate that defect in cell shape caused by Δ*flhE* is not causing OM pore formation. Left side: signal observed in a WT treated with 70% EtOH (positive control), WT, Δ*flhE* and P_{synC}-*flhDC* background. Right side: exemplary pictures of P_{synC}-*flhDC* Δ*flhE* background. Cell with aberrant morphology display no SYTOX green signal. Scale bar = 2 µm. **e** Formation of minicells in Δ*flhE* background. Microfluidic experiment (CellAsic ONIX, Merck) of P_{synC}-*flhDC* (yellow, mNeonGreen) and P_{synC}-*flhDC* Δ*flhE* (cyan, mCerulean) expressing fluorescent proteins from a constitutive promoter carried on a vector. Δ*flhE* strain display a growth defect phenotype. White square represents the field of view displayed in Fig. 2c. Scale bar = 10 µm. Data shown are from one representative experiment.

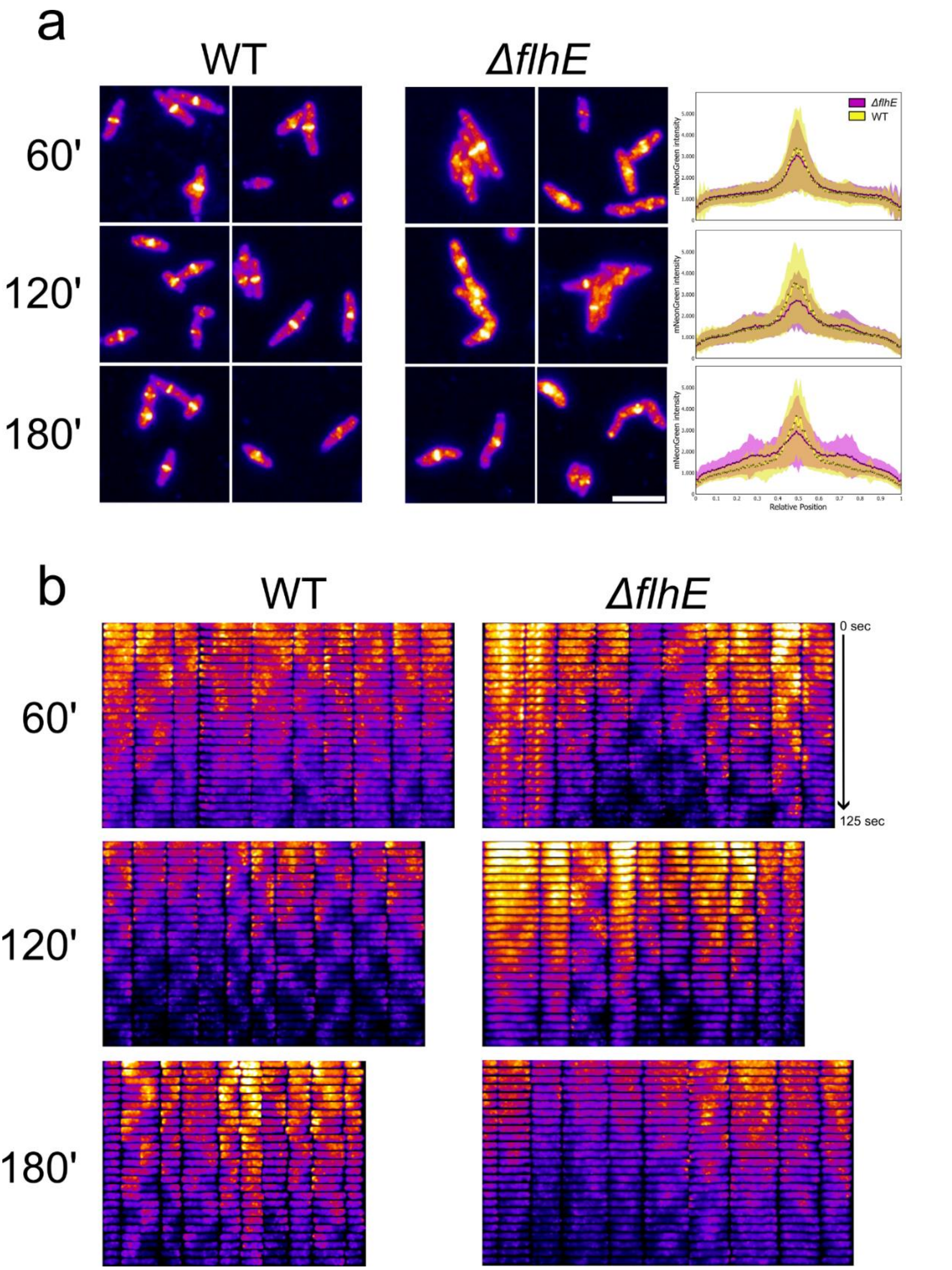

Fig. S6: Growth defect in Δ*flhE* Δ*flgHI* mutant perturbate ZapA localisation and MinD oscillations. **a** Time course TIRF experiment of mNeonGreen-ZapA in Δ*flgHI* and Δ*flhE* Δ*flgHI* background shows ectopic localisation of ZapA rings. Left panel: exemplary images of mNeonGreen-ZapA. Scale bar = 5 µm. Right panel: measurement of fluorescence intensity on the medial profile (1000 bins) of at least 272 cells analysed per timepoint and strain (WT

in yellow, Δ*flhE* mutant in magenta). Overtime, the fluorescence intensity is shifting from the centre to the poles in the Δ*flhE* Δ*flgHI* background. Source data are provided as a Source Data file. Data shown are from one representative experiment and have been independently replicated in at least one preliminary experiment. **b** Time course TIRF experiment of mNeonGreen-MinD in Δ*flgHI* and Δ*flhE* Δ*flgHI* background. Cells were imaged 60, 120 and 180 min after AnTc induction of P_{tetA}-*flhDC*. A time-lapse was acquired with 5 sec intervals between each picture. Cells were aligned in a demograph using MicrobeJ. MinD oscillations is decreased in Δ*flhE* Δ*flgHI* background relative to Δ*flgHI* background.

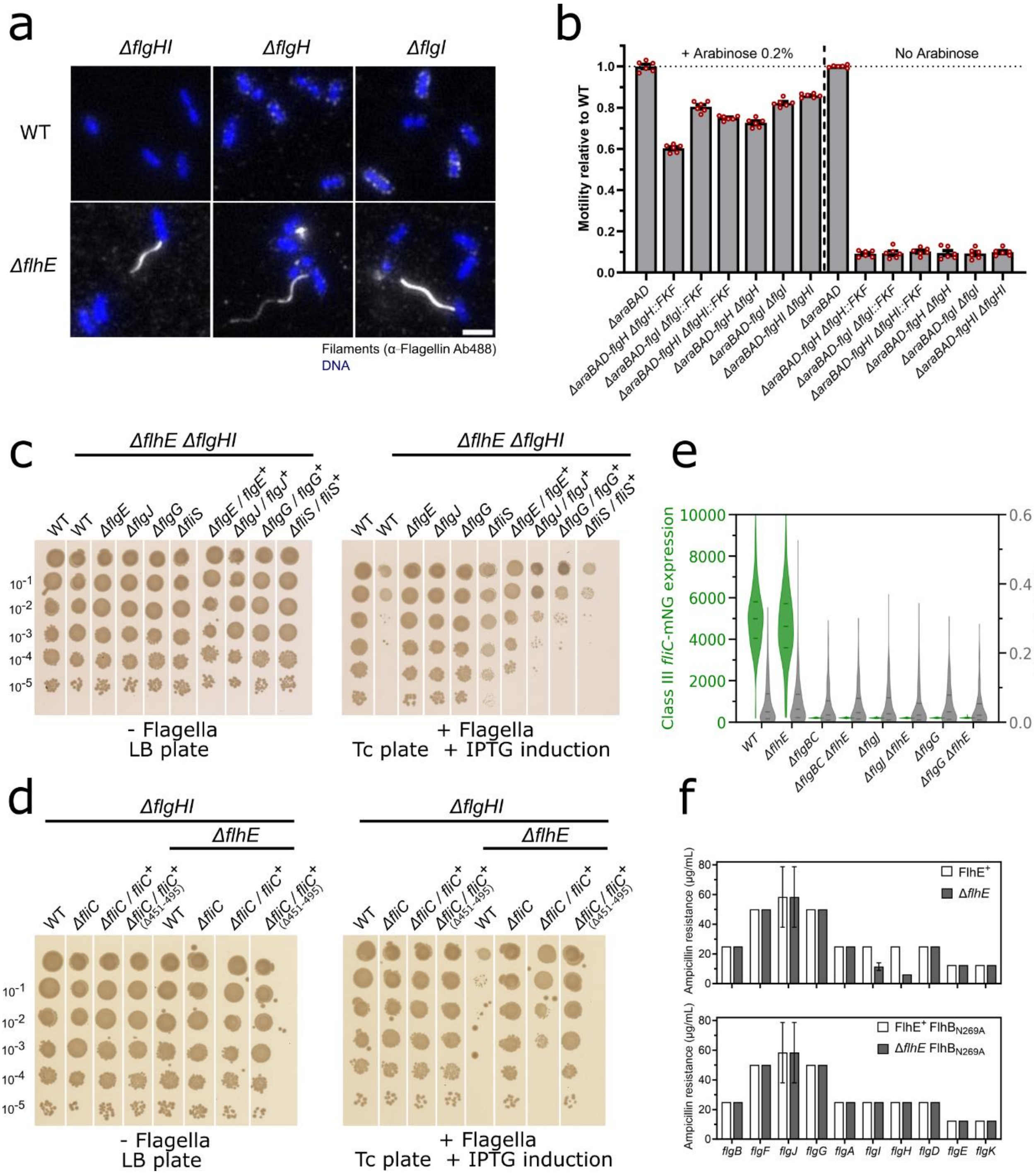

Fig. S7: Phenotype of Δ*flgHI* Δ*flhE* background in present of other deletion of the fT3SS structure. **a** Immunostaining of flagellar filament reveals that Δ*flhE* Δ*flgHI*/Δ*flgH*/Δ*flgI* strain can assemble filaments. Immunostaining were performed with primary anti-FliC antibodies and secondary antibodies coupled to Alexa488 (white). DNA staining was performed with Fluoroshield with DAPI (blue). Scale bar = 2 µm. **b** Complementation of Δ*flgHI*::FKF and Δ*flgHI* clean deletion using chromosomal *araBAD* locus showed a mild polar effect on the *flg* operon. Motility was complemented between 60 to 85%. Motility tests in swimming agar were performed at 37°C. Biological duplicates were performed with 3 technical replicates, and motility of the various strains was calculated relative to WT for each plate. **c** Spot-assay of

∆*flhE* ∆*flgHI* strains coupled with a deletion of the hook (∆*flgE),* the rod (∆*flgJ / ∆flgG),* or of the flagellin chaperone *(∆fliS).* Flagellar system is only expressed in presence of tetracycline inducer (+Flagella, Tc plate). In absence of inducer (-Flagella, LB plate), no growth defect can be observed for any of the strains tested. Deletion were complemented *in trans* from an IPTG inducible vector to exclude polar effect. **d** Spot-assay of ∆*flhE* ∆*flgHI* strains coupled with a deletion of the flagellin (∆*fliC).* ∆*fliC* was complemented *in trans* from a vector expressing the full-length FliC or a truncated C-terminal version of FliC (∆aa451-495*)* unable to assemble flagellar filaments. No growth defect could be observed in absence of full-length FliC. Data shown are from one representative experiment and have been independently replicated in at least two independent experiment. **e** Expression of class III reporter *fliC-mneongreen* transcriptional fusion (green violin plot) and cell curvature (grey violin plot) in WT and ∆*flhE* background (*flgHI*$^{+}$)*,* combined with a deletion of rod component *(∆flgBC, ∆flgJ, ∆flgG)* 180 min after induction of flagellar system with AnTc. No class III expression can be observed in ∆*flhE* in absence of the rod components*.* Cell curvature is restored to WT in absence of the rod. At least 300 cells per strain were analysed. **f** Secretion of FliK monitored using a FliK-bla reporter in ∆*flhE* strain associated with a deletion of the rod or the PL-rings. Secretion of FliK in the periplasm is detected by ampicillin resistance from the Bla fusion. No differences in FliK secretion can be observed between WT and ∆*flhE* strain for any of the deletion, except deletion of the P/L-rings (∆*flgH / ∆flgI).* Introduction of a $FlhB_{N269A}$ mutation, unable to undergo the substrate specificity switch, restore the WT levels of FliK secretion (N = 6). Source data are provided as a Source Data file.

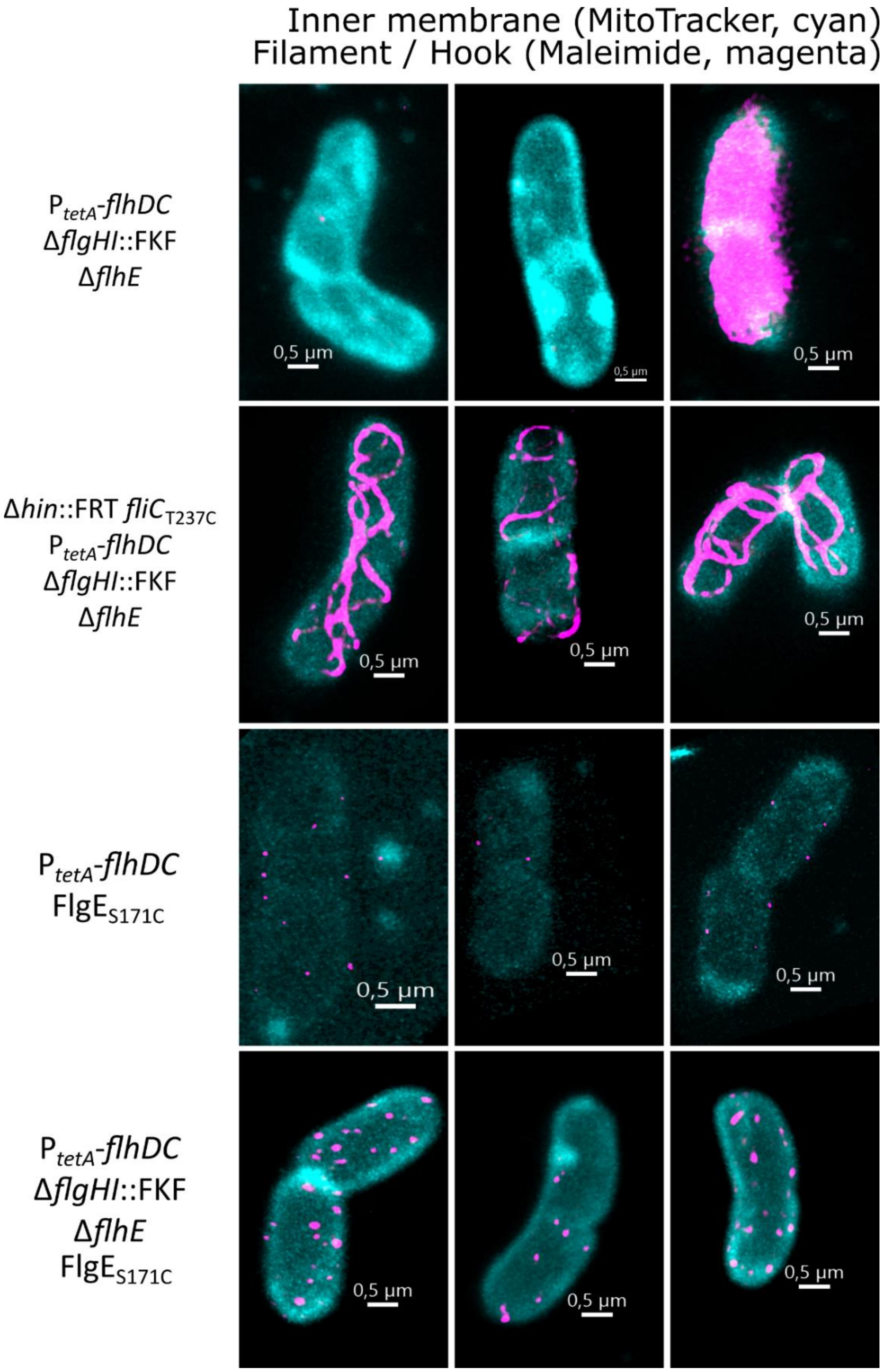

Fig. S8: Representative 3D images of maleimide staining of the periplasmic flagellar filament and hooks. Cells were stained with maleimide STAR RED (magenta) and MitoTracker Green (cyan) and imaged using STED super-resolution microscopy, with Z-stack intervals of 80 nm. Scale bar = 0.5 µm. Data shown are from one representative experiment and have been independently replicated in at least one preliminary experiment.

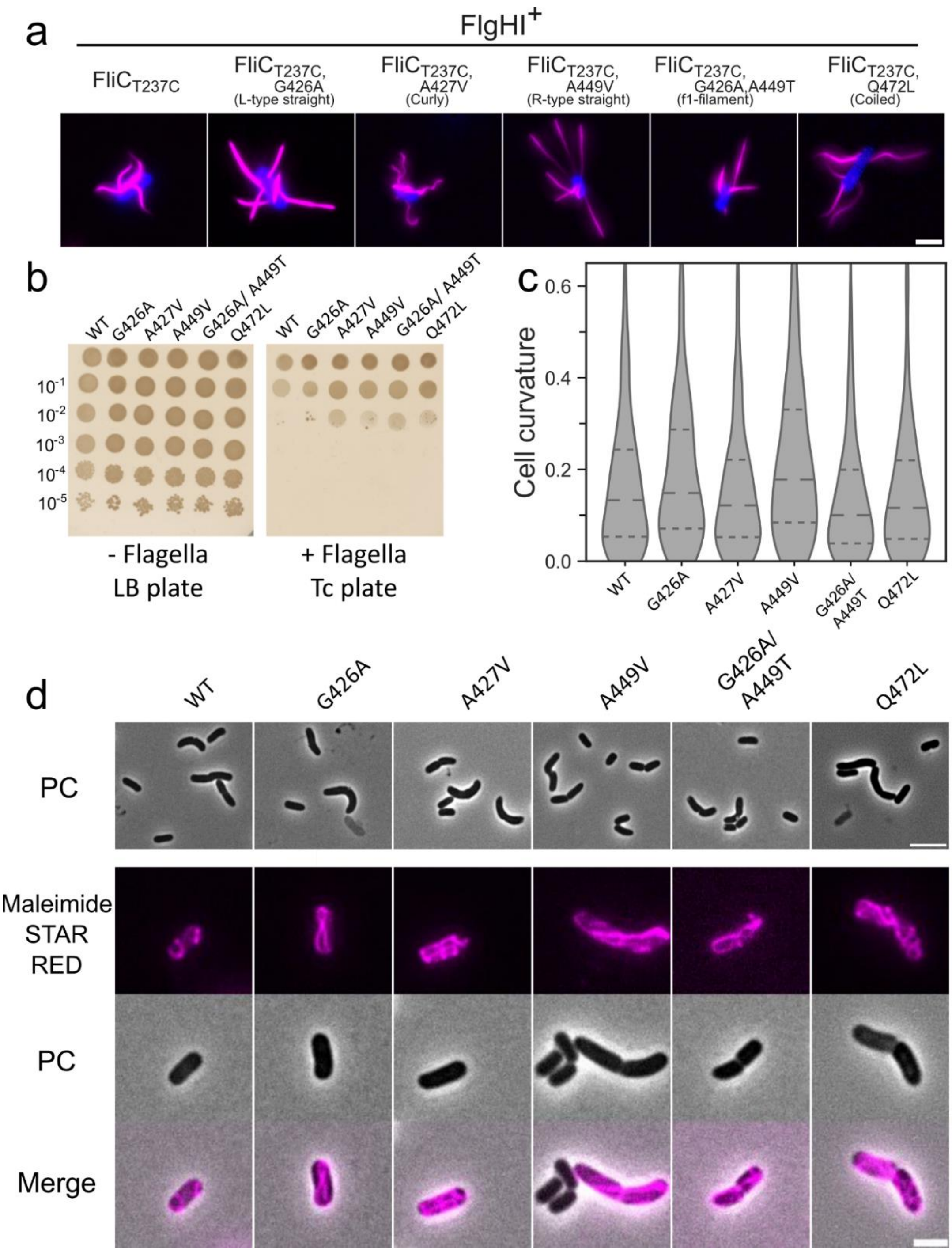

Fig. S9: Morphology of the flagellar filament does not impact the cell curvature. **a** Maleimide staining (magenta) of $FliC_{T237C}$ with secondary point mutations changing the morphology of the filament from supercoiled to straight / curly / coiled / f1. Staining was performed in $FlgHI^{+}$ background, where filaments are assembled extracellularly. DNA staining with DAPI is represented in blue. Scale bar = 2 µm. **b** Spot-assay of Δ*flhE* Δ*flgHI* strains coupled with the FliC point mutants described in **a**. The growth defect observed is similar between all FliC point mutants. **c** Cell curvature analysis of the strains described in **b** observed by phase contrast microscopy. At least 400 cells per strain were analysed. Source data are provided as a Source

Data file. **d** Phase contrast and maleimide staining (magenta) of $FliC_{T237C}$ variants in ∆*flhE* ∆*flgHI background* observed by epifluorescence microscopy. Cells with curved shape can be observed independent of the FliC variant. Scale bar = 2 µm. Data shown are from one representative experiment.

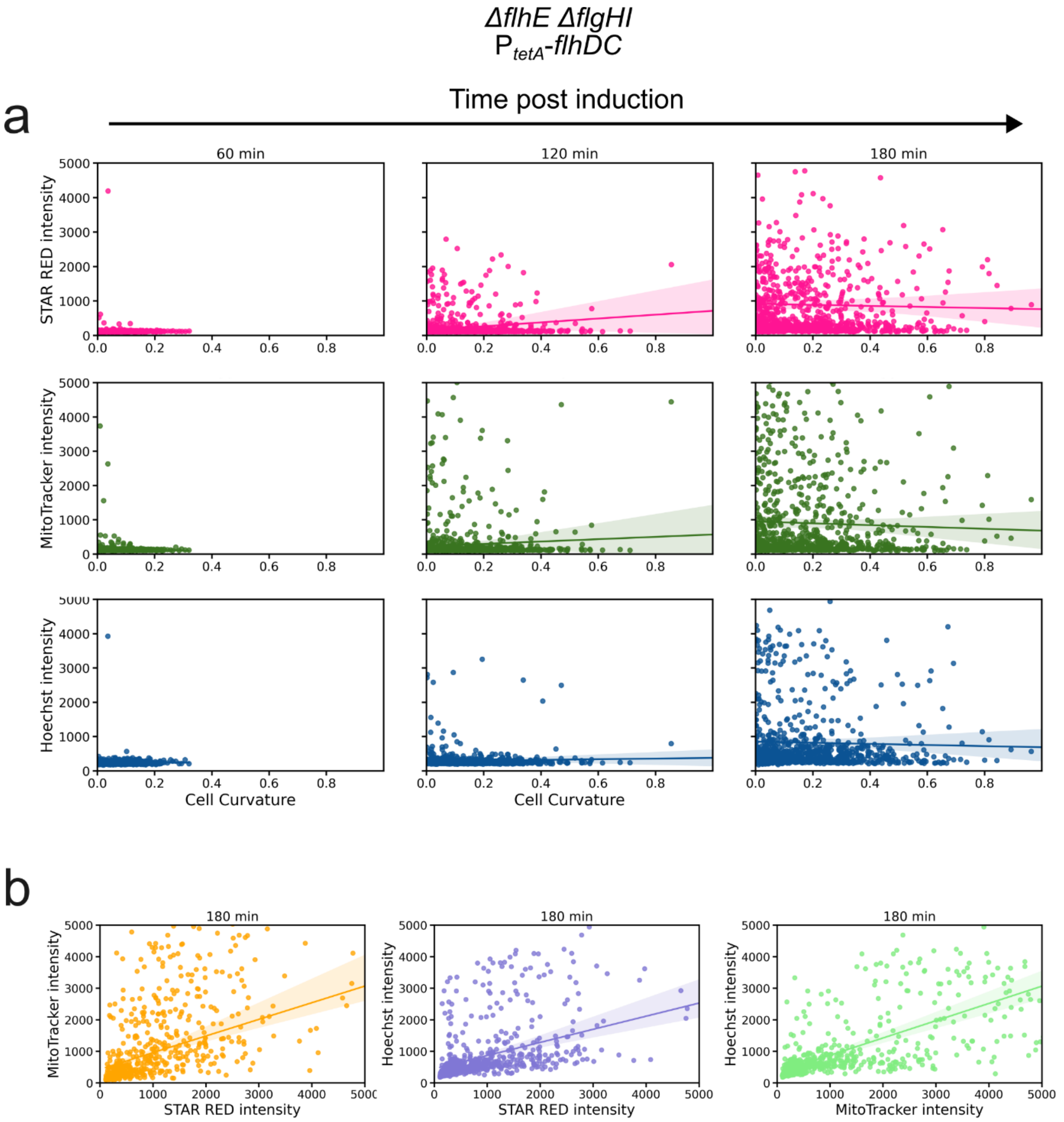

Fig. S10: Correlation between cell curvature and membrane staining. **a** Upper panel: STAR RED maleimide (magenta) intensity plotted against cell curvature; middle panel: MitoTracker Green (IM, green) intensity plotted against cell curvature; lower panel: Hoechst intensity (blue, DNA staining) plotted against cell curvature. For each staining, the increase in intensity is not directly correlated to the increase in cell curvature. **b** Dye staining relative plot. Left: STAR RED intensity plotted against MitoTracker Green; middle: STAR RED intensity plotted against Hoechst intensity; right: MitoTracker intensity plotted against Hoechst intensity. For each plot, a correlation can be observed as intensity of the different dyes increase. Independent biological triplicates were performed, and at least 283 cells were analysed per timepoint. Source data are provided as a Source Data file.

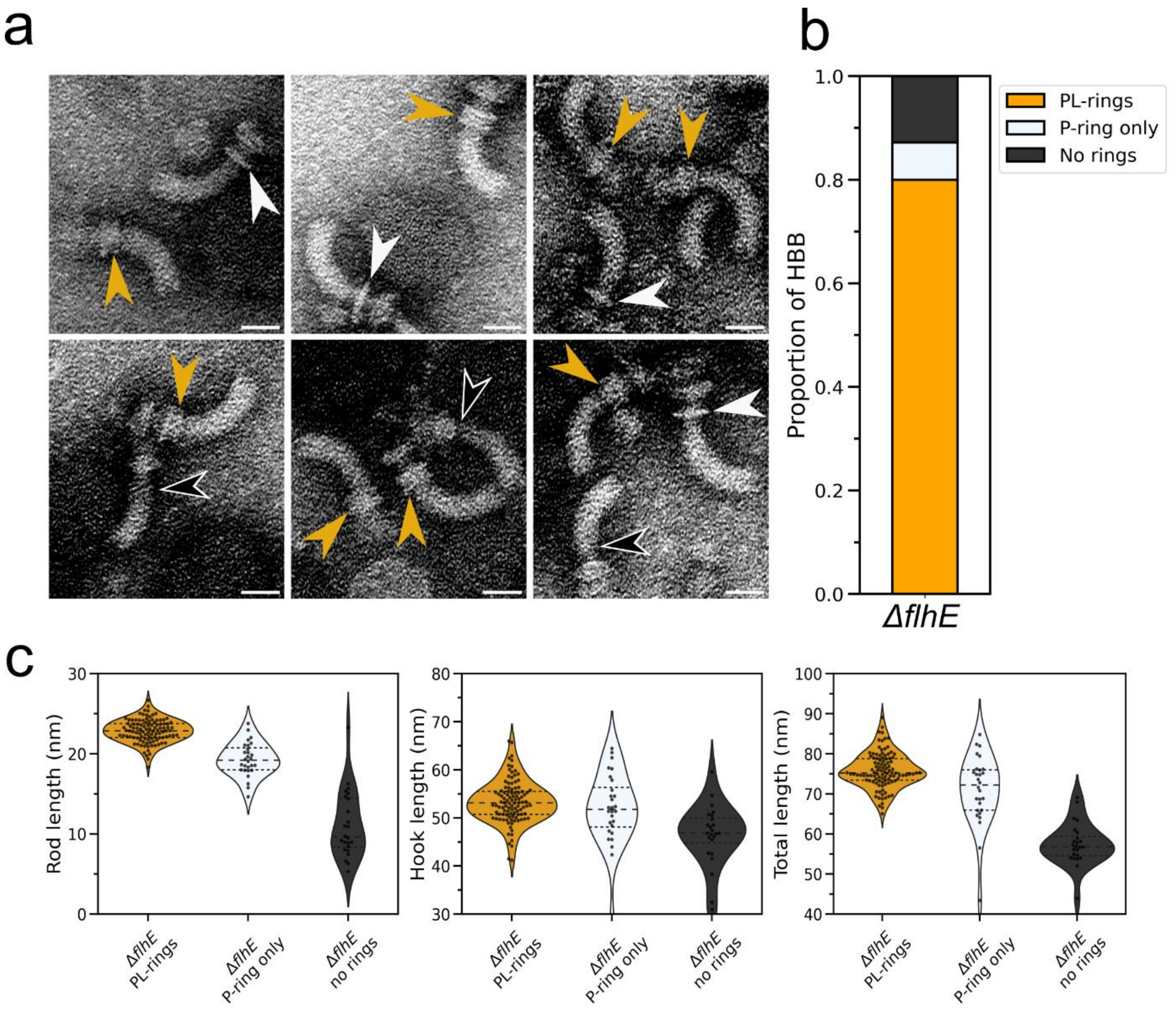

Fig. S11: Δ*flhE* assemble rods lacking P and L-rings. **a** Exemplary pictures of HBB purified from Δ*flhE,* observed by TEM. Most of the HBB include in their structure both PL-rings (orange arrow), while some were either missing the L-ring (P-ring only, white arrows) or both rings (no rings, black arrow). Scale bar = 25 nm. **b** Proportion of HBB with both rings, P-ring only or no rings. A total of 305 HBB were manually counted for presence of the rings. **c** Rod, hook and total (rod + hook) length for HBB from Δ*flhE* HBB purification in presence or absence of the rings. Absence of both rings is resulting in a drastic decrease of the rod length. Source data are provided as a Source Data file.

Table S1: Strains used in this study

Name	Genotype	Reference
TH437	LT2	J. Roth
TH12483	Δ*flhE*7404	Lab collection
EM9060	Δ*flhE7404*<br>/ pEM8913 (pKH70-P$_{rpsM}$-*flhE*)	This study
EM11563	Δ*flhE*23076::FCF	This study
EM12322	Δ*flhE*23076::FRT	This study
EM11990	Δ*flhE*7404 Δ*fliP*23466::FRT	This study
EM11991	Δ*flhE*7404 Δ*fliPQR*23467::FRT	This study
EM11988	Δ*flhE*7404 Δ*flhA*23464::FRT	This study
EM11987	Δ*flhE*7404 Δ*flhB*23463::FRT	This study
EM11989	Δ*flhE*7404 Δ*flhBA*23465::FRT	This study
EM11028	Δ*flhE*7404 Δ*flgBC*6557	This study
EM8344	Δ*flhE*7404 Δ*flgE*22964::FCF	This study
EM11243	Δ*flhE*7404 Δ*hin*5717::FRT Δ*flgKL*5739::FKF	This study
EM12631	Δ*flhE*7404 Δ*flgKL*7770	This study
EM11557	Δ*flhE*7404 Δ*fliC*7861::FRT Δ*hin*-5717::FCF (fliC^{ON})	This study
EM8498	Δ*flhE*7404 Δ*hin*-5717::FRT (fliC^{ON})	This study
EM11556	Δ*flhE*7404 Δ*fliC*7861::FRT Δ*hin*-5718::FCF (fljB^{ON})	This study
EM8397	Δ*flhE*7404 *flhB*23151$_{N269A}$	This study
EM12336	*fliK*6620(Δ aa 248-298) Δ*flhE*23076::FCF	This study
EM12337	Δ*hin*-5717::FRT *flgE*6506$_{S171C}$ Δ*fliK*6137 (clean deletion) Δ*flhE*23076::FCF	This study
EM8493	Δ*flhE*7404 Δ*fliS*5728::FRT	This study
EM12379	Δ*hin*-5717::FRT *fliC*5936(FliC-GAGAGAGA-GFPmut2) Δ*flhE*23076::FCF	This study
EM12130	Δ*flhE*23076::FCF Δ*motAB*::tetRA (Δaa R90-F63)	This study
EM12516	Δ*motAB* (leaving first and last 15 bp) Δ*flhE*23076::FCF	This study
EM12515	*motA*5461::*mudJ* Δ*flhE*23076::FCF	This study
EM12487	*motA*$_{M206A}$ Δ*flhE*23076::FCF	This study
EM12489	*motB*$_{D33A}$ Δ*flhE*23076::FCF	This study
EM12008	Δ*flhE*23076::FCF Δ*cheY* 22371::FKF	This study
TH9677	Δ*hin*-5717::FRT *flgE*6506$_{S171C}$	Lab collection
EM10163	Δ*hin*-5717::FRT *flgE*6506$_{S171C}$ Δ*flhE*23076::FCF	This study
EM9659	Δ*hin*-5717::FRT *flgE*6506$_{S171C}$ P$_{proC}$-*flhDC*23252 (Δbp -598 to AUG of *flhD)*	Lab collection
EM10697	Δ*hin*-5717::FRT *flgE*6506$_{S171C}$ P$_{proC}$-*flhDC*23252 Δ*flhE*23076::FCF	This study
EM12325	Δ*hin*-5717::FRT *flgE*6506$_{S171C}$ P$_{proC}$-*flhDC*23252 Δ*flhE*23076::FRT	This study
EM12439	Δ*hin*-5717::FRT *flgE*6506$_{S171C}$ P$_{proC}$-*flhDC*23252<br>/ pEM8913 (pKH70-P$_{rpsM}$-*flhE*)	This study
EM12440	Δ*hin*-5717::FRT *flgE*6506$_{S171C}$ P$_{proC}$-*flhDC*23252 Δ*flhE*23076::FRT<br>/ pEM8913 (pKH70-P$_{rpsM}$-*flhE*)	This study
EM12765	Δ*hin*-5717::FRT *flgE*6506$_{S171C}$ P$_{proC}$-*flhDC*23252 Δ*fliS*5728::FKF	This study
EM12766	Δ*hin*-5717::FRT *flgE*6506$_{S171C}$ P$_{proC}$-*flhDC*23252 Δ*flhE*23076::FRT Δ*fliS*5728::FKF	This study
EM13389	Δ*hin*-5717::FRT *flgE*6506$_{S171C}$ P$_{proC}$-*flhDC*23252 Δ*flgKL*5739::FKF	This study
EM12366	Δ*hin*-5717::FRT *flgE*6506$_{S171C}$ P$_{proC}$-*flhDC*23252 Δ*flhE*23076::FCF Δ*flgKL*5739::FKF	This study
EM13681	Δ*hin*-5717::FRT *flgE*6506$_{S171C}$ P$_{proC}$-*flhDC*23252 Δ*flgKL*5739::FKF Δ*araBAD*1404::*flgKL*	This study
EM13744	Δ*hin*-5717::FRT *flgE*6506$_{S171C}$ P$_{proC}$-*flhDC*23252 Δ*flgKL*5739::FKF<br>Δ*araBAD*1404::*flgKL* Δ*flhE*23076::FCF	This study
EM9662	Δ*hin*-5717::FRT *flgE*6506$_{S171C}$ P$_{pro1}$-*flhDC*23252 (Δbp -598 to AUG of *flhD)*	Lab collection
EM9661	Δ*hin*-5717::FRT *flgE*6506$_{S171C}$ P$_{proA}$-*flhDC*23252 (Δbp -598 to AUG of *flhD)*	Lab collection
EM9660	Δ*hin*-5717::FRT *flgE*6506$_{S171C}$ P$_{proB}$-*flhDC*23252 (Δbp -598 to AUG of *flhD)*	Lab collection
EM10165	Δ*hin*-5717::FRT *flgE*6506$_{S171C}$ P$_{pro1}$-*flhDC*23252 Δ*flhE*23076::FCF	This study
EM10164	Δ*hin*-5717::FRT *flgE*6506$_{S171C}$ P$_{proA}$-*flhDC*23252 Δ*flhE*23076::FCF	This study
EM10698	Δ*hin*-5717::FRT *flgE*6506$_{S171C}$ P$_{proB}$-*flhDC*23252 Δ*flhE*23076::FCF	This study
EM13743	Δ*hin*-5717::FRT *flgE*6506$_{S171C}$ P$_{proC}$-*flhDC*23252<br>Δ*araBAD*1405::*fliC*23546-GAGAGAGA-GFPmut2 Δ*flhE*23076::FCF	This study
EM8250	*fliG*22799 (mNeonGreen-FliG, N-terminal) *flgE*6506$_{S171C}$	Lab collection, [1]
EM11018	*fliG*22799 *flgE*6506$_{S171C}$ Δ*flhE*23076::FCF	This study
EM15307	*fliG*22799 *flgE*6506$_{S171C}$ P$_{tetA}$*flhDC*5451::Tn10dTc[del-25]	This study
EM15308	*fliG*22799 *flgE*6506$_{S171C}$ Δ*flhE*23076::FCF P$_{tetA}$*flhDC*5451::Tn10dTc[del-25]	This study
EM12501	Δ*hin*-5717::FRT *flgE*6506$_{S171C}$ P$_{proC}$-*flhDC*23252 *fliG*22799	This study
EM12844	Δ*hin*-5717::FRT *flgE*6506$_{S171C}$ P$_{proC}$-*flhDC*23252 *fliG*22799 Δ*flhE*23076::FCF	This study
EM12367	DH5alpha / pXY027 (pCA24N-*ftsZ-GFP*)	Addgene Plasmid #98915 [2]
EM12437	Δ*hin*-5717::FRT *flgE*6506$_{S171C}$ P$_{proC}$-*flhDC*23252<br>/ pXY027 (pCA24N-*ftsZ-GFP*)	This study
EM12438	Δ*hin*-5717::FRT *flgE*6506$_{S171C}$ P$_{proC}$-*flhDC*23252 Δ*flhE*23076::FRT<br>/ pXY027 (pCA24N-*ftsZ-gfp*)	This study

EM12594	Δ*hin*-5717::FRT *flgE*6506$_{S171C}$ P_{proC}-*flhDC*23252 / pEM12582 (pCA24N-*mreB*(G228)-*mneongreen*-*mreB*(D229))	This study
EM12597	Δ*hin*-5717::FRT *flgE*6506$_{S171C}$ P_{proC}-*flhDC*23252 Δ*flhE*23076::FRT / pEM12582 (pCA24N-*mreB*(G228)-*mneongreen*-*mreB*(D229))	This study
EM12362	Δ*hin*-5717::FRT *flgE*6506$_{S171C}$ P_{proC}-*flhDC*23252 / pEM8731 (pKH70-P_{rpsM}-*mneongreen*)	This study
EM12550	Δ*hin*-5717::FRT *flgE*6506$_{S171C}$ P_{proC}-*flhDC*23252 Δ*flhE*23076::FRT / pEM8313 (pKH70-P_{rpsM}-*mcerulean*)	This study
TH3730	P_{tetA}*flhDC*5451::Tn10dTc[del-25]	Lab collection
EM10212	P_{tetA}*flhDC*5451::Tn10dTc[del-25] Δ*flhE*23076::FCF	This study
TH14357	Δ*flgBC*6557 P_{tetA}*flhDC*5451::Tn10dTc[del-25]	Lab collection
EM14564	Δ*flgBC*6557 P_{tetA}*flhDC*5451::Tn10dTc[del-25] Δ*flhE*23076::FCF	This study
EM14079	Δ*flgHI*958 P_{tetA}*flhDC*5451::Tn10dTc[del-25]	This study
EM14216	Δ*flgHI*958 P_{tetA}*flhDC*5451::Tn10dTc[del-25] Δ*flhE*23076::FCF	This study
EM14242	Δ*flgH*7662 P_{tetA}*flhDC*5451::Tn10dTc[del-25]	This study
EM14217	Δ*flgH*7662 P_{tetA}*flhDC*5451::Tn10dTc[del-25] Δ*flhE*23076::FCF	This study
EM14243	Δ*flgI*7663 P_{tetA}*flhDC*5451::Tn10dTc[del-25]	This study
EM14218	Δ*flgI*7663 P_{tetA}*flhDC*5451::Tn10dTc[del-25] Δ*flhE*23076::FCF	This study
EM9357	*fliC*23165-*mneongreen* (transcriptional fusion, insertion RBS (P_{rpsM}) in 3'UTR *fliC*) P_{tetA}*flhDC*5451::Tn10dTc[del-25] Δ*hin*-5717::FRT	This study
EM13604	*fliC*23165-*mneongreen* P_{tetA}*flhDC*5451::Tn10dTc[del-25] Δ*hin*-5717::FRT Δ*flhE*23076::FRT	This study
EM14435	*fliC*23165-*mneongreen* P_{tetA}*flhDC*5451::Tn10dTc[del-25] Δ*hin*-5717::FRT Δ*flgHI*23618::FKF	This study
EM14436	*fliC*23165-*mneongreen* P_{tetA}*flhDC*5451::Tn10dTc[del-25] Δ*hin*-5717::FRT Δ*flhE*23076::FRT Δ*flgHI*23618::FKF	This study
EM15573	*fliC*23165-*mneongreen* P_{tetA}*flhDC*5451::Tn10dTc[del-25] Δ*hin*-5717::FRT Δ*flgHI*23618::FRT / pTrc99AFF4	This study
EM15574	*fliC*23165-*mneongreen* P_{tetA}*flhDC*5451::Tn10dTc[del-25] Δ*hin*-5717::FRT Δ*flhE*23076::FRT Δ*flgHI*23618::FRT / pTrc99AFF4	This study
EM15638	*fliC*23165-*mneongreen* P_{tetA}*flhDC*5451::Tn10dTc[del-25] Δ*hin*-5717::FRT Δ*flgHI*23618::FRT Δ*flgE*22964::FRT / pTrc99AFF4	This study
EM15639	*fliC*23165-*mneongreen* P_{tetA}*flhDC*5451::Tn10dTc[del-25] Δ*hin*-5717::FRT Δ*flhE*23076::FRT Δ*flgHI*23618::FRT Δ*flgE*22964::FRT / pTrc99AFF4-*flgE*$^{+}$	This study
EM14432	Δ*araBAD*1001::*flgH* Δ*flgH*23616::FKF	This study
EM14433	Δ*araBAD*1002::*flgI* Δ*flgI*23617::FKF	This study
EM14434	Δ*araBAD*941::*flgHI* Δ*flgHI*23618::FKF	This study
EM14463	Δ*flgH*7662 Δ*araBAD*1001::*flgH*	This study
EM14464	Δ*flgI*7663 Δ*araBAD*1002::*flgI*	This study
EM14465	Δ*flgHI*958 Δ*araBAD*941::*flgHI*	This study
EM808	Δ*araBAD*1005::FRT	Lab collection
EM14678	Δ*flgHI*958 P_{tetA}*flhDC*5451::Tn10dTc[del-25] / pTrc99AFF4	This study
EM14676	Δ*flgHI*958 P_{tetA}*flhDC*5451::Tn10dTc[del-25] Δ*flhE*23076::FCF / pTrc99AFF4	This study
EM14750	Δ*flgHI*958 P_{tetA}*flhDC*5451::Tn10dTc[del-25] Δ*flhE*23076::FCF Δ*flgE*23643::FKF / pTrc99AFF4	This study
EM14751	Δ*flgHI*958 P_{tetA}*flhDC*5451::Tn10dTc[del-25] Δ*flhE*23076::FCF Δ*flgE*23643::FKF / pTrc99AFF4-*flgE*$^{+}$	This study
EM14752	Δ*flgHI*958 P_{tetA}*flhDC*5451::Tn10dTc[del-25] Δ*flhE*23076::FCF Δ*flgJ*23644::FKF / pTrc99AFF4	This study
EM14753	Δ*flgHI*958 P_{tetA}*flhDC*5451::Tn10dTc[del-25] Δ*flhE*23076::FCF Δ*flgJ*23644::FKF / pTrc99AFF4-*flgJ*$^{+}$	This study
EM14754	Δ*flgHI*958 P_{tetA}*flhDC*5451::Tn10dTc[del-25] Δ*flhE*23076::FCF Δ*flgG*23645::FKF / pTrc99AFF4	This study
EM14755	Δ*flgHI*958 P_{tetA}*flhDC*5451::Tn10dTc[del-25] Δ*flhE*23076::FCF Δ*flgG*23645::FKF / pTrc99AFF4-*flgG*$^{+}$	This study
EM14756	Δ*flgHI*958 P_{tetA}*flhDC*5451::Tn10dTc[del-25] Δ*flhE*23076::FCF Δ*fliS*5720::FKF / pTrc99AFF4	
EM14757	Δ*flgHI*958 P_{tetA}*flhDC*5451::Tn10dTc[del-25] Δ*flhE*23076::FCF Δ*fliS*5720::FKF / pTrc99AFF4-*fliS*$^{+}$	This study
EM14758	Δ*fliC*7861::FRT Δ*hin*-5717::FCF (fliC^{ON}) P_{tetA}*flhDC*5451::Tn10dTc[del-25] Δ*flgH*23616::FKF / pTrc99AFF4	This study
EM14759	Δ*fliC*7861::FRT Δ*hin*-5717::FCF (fliC^{ON}) P_{tetA}*flhDC*5451::Tn10dTc[del-25] Δ*flgH*23616::FKF / pTrc99A FF4-*fliC*$^{+}$	This study
EM14760	Δ*fliC*7861::FRT Δ*hin*-5717::FCF (fliC^{ON}) P_{tetA}*flhDC*5451::Tn10dTc[del-25] Δ*flgH*23616::FKF / pTrc99A FF4-*fliC* (Δ aa 451-495)	This study
EM14761	Δ*flhE*7404 Δ*fliC*7861::FRT Δ*hin*-5717::FCF (fliC^{ON}) P_{tetA}*flhDC*5451::Tn10dTc[del-25] Δ*flgH*23616::FKF	This study

	/ pTrc99AFF4	
EM14762	Δ*flhE*7404 Δ*fliC*7861::FRT Δ*hin*-5717::FCF (fliC^{ON}) P_{tetA}*flhDC*5451::Tn10dTc[del-25] Δ*flgH*23616::FKF / pTrc99A FF4-*fliC*$^{+}$	This study
EM14763	Δ*flhE*7404 Δ*fliC*7861::FRT Δ*hin*-5717::FCF (fliC^{ON}) P_{tetA}*flhDC*5451::Tn10dTc[del-25] Δ*flgH*23616::FKF / pTrc99A FF4-*fliC* (Δ aa 451-495)	This study
EM15551	*fliC*23165-*mneongreen* P_{tetA}*flhDC*5451::Tn10dTc[del-25] Δ*hin*-5717::FRT Δ*flhE*23076::FRT Δ*flgBC*6557	This study
EM15552	*fliC*23165-*mneongreen* P_{tetA}*flhDC*5451::Tn10dTc[del-25] Δ*hin*-5717::FRT Δ*flgBC*6557	This study
EM15553	*fliC*23165-*mneongreen* P_{tetA}*flhDC*5451::Tn10dTc[del-25] Δ*hin*-5717::FRT Δ*flhE*23076::FRT Δ*flgJ*7600	This study
EM15554	*fliC*23165-*mneongreen* P_{tetA}*flhDC*5451::Tn10dTc[del-25] Δ*hin*-5717::FRT Δ*flgJ*7600	This study
EM15555	*fliC*23165-*mneongreen* P_{tetA}*flhDC*5451::Tn10dTc[del-25] Δ*hin*-5717::FRT Δ*flhE*23076::FRT Δ*flgG*7661	This study
EM15556	*fliC*23165-*mneongreen* P_{tetA}*flhDC*5451::Tn10dTc[del-25] Δ*hin*-5717::FRT Δ*flgG*7661	This study
EM14439	Δ*hin*-5717::FRT *fliC*5600$_{T237C}$ P_{tetA}*flhDC*5451::Tn10dTc[del-25] Δ*flgHI*23618::FKF	This study
EM14440	Δ*hin*-5717::FRT *fliC*5600$_{T237C}$ Δ*flhE*7404 P_{tetA}*flhDC*5451::Tn10dTc[del-25] Δ*flgHI*23618::FKF	This study
EM4872	Δ*hin*-5717::FRT *flgE*6506$_{S171C}$ P_{tetA}*flhDC*5451::Tn10dTc[del-25]	Lab collection
EM14769	Δ*hin*-5717::FRT *flgE*6506$_{S171C}$ P_{tetA}*flhDC*5451::Tn10dTc[del-25] Δ*flhE*23076::FRT Δ*flgHI*23618::FKF	This study
EM2046	Δ*hin*-5717::FRT *fliC*5600$_{T237C}$ P_{tetA}*flhDC*5451::Tn10dTc[del-25]	Lab collection
EM9889	Δ*hin*-5717::FRT *fliC*23284$_{T237C, G426A}$ P_{tetA}*flhDC*5451::Tn10dTc[del-25]	This study
EM9890	Δ*hin*-5717::FRT *fliC*23285$_{T237C, A427V}$ P_{tetA}*flhDC*5451::Tn10dTc[del-25]	This study
EM9891	Δ*hin*-5717::FRT *fliC*23286$_{T237C, A449V}$ P_{tetA}*flhDC*5451::Tn10dTc[del-25]	This study
EM9892	Δ*hin*-5717::FRT *fliC*23287$_{T237C, G426A, A449T}$ P_{tetA}*flhDC*5451::Tn10dTc[del-25]	This study
EM9893	Δ*hin*-5717::FRT *fliC*23288$_{T237C, Q472L}$ P_{tetA}*flhDC*5451::Tn10dTc[del-25]	This study
EM15686	Δ*hin*-5717::FRT *fliC*5600$_{T237C}$ P_{tetA}*flhDC*5451::Tn10dTc[del-25] Δ*flgHI*23618::FKF Δ*flhE*23076::FCF	This study
EM15706	Δ*hin*-5717::FRT *fliC*23284$_{T237C, G426A}$ P_{tetA}*flhDC*5451::Tn10dTc[del-25] Δ*flgHI*23618::FKF Δ*flhE*23076::FCF	This study
EM15707	Δ*hin*-5717::FRT *fliC*23285$_{T237C, A427V}$ P_{tetA}*flhDC*5451::Tn10dTc[del-25] Δ*flgHI*23618::FKF Δ*flhE*23076::FCF	This study
EM15708	Δ*hin*-5717::FRT *fliC*23286$_{T237C, A449V}$ P_{tetA}*flhDC*5451::Tn10dTc[del-25] Δ*flgHI*23618::FKF Δ*flhE*23076::FCF	This study
EM15709	Δ*hin*-5717::FRT *fliC*23287$_{T237C, G426A, A449T}$ P_{tetA}*flhDC*5451::Tn10dTc[del-25] Δ*flgHI*23618::FKF Δ*flhE*23076::FCF	This study
EM15710	Δ*hin*-5717::FRT *fliC*23288$_{T237C, Q472L}$ P_{tetA}*flhDC*5451::Tn10dTc[del-25] Δ*flgHI*23618::FKF Δ*flhE*23076::FCF	This study
EM15447	Δ*flgHI*958 P_{tetA}*flhDC*5451::Tn10dTc[del-25] *mneongreen-zapA* (N-ter, no linker)	This study
EM15505	Δ*flgHI*958 P_{tetA}*flhDC*5451::Tn10dTc[del-25] *mneongreen-zapA*	This study
EM15918	Δ*flgHI*958 P_{tetA}*flhDC*5451::Tn10dTc[del-25] *mneongreen-minD* (N-ter, SAGASA linker)	This study
EM15974	Δ*flgHI*958 P_{tetA}*flhDC*5451::Tn10dTc[del-25] *mneongreen-minD*	This study

Table S2: Strains used for FliK-Bla assay

Name	Genotype	Reference
EM11563	Δ*flhE*23076::FCF	This study
EM12322	Δ*flhE*23076::FRT	Lab collection
TH13359	*fliK7582::bla* (before stop)	Lab collection
TH13807	*fliK7582::bla* Δ*flgA7656*(D6-214(of 219))	Lab collection
TH13808	*fliK7582::bla* Δ*flgB7657*(D6-133(of 138))	Lab collection
TH13809	*fliK7582::bla* Δ*flgC7658*(D6-129(of 134))	Lab collection
TH13810	*fliK7582::bla* Δ*flgD6540*(D2-220(of 232))	Lab collection
TH13811	*fliK7582::bla* Δ*flgE7659*(D6-398(of 403))	Lab collection
TH13812	*fliK7582::bla* Δ*flgF7660*(D6-246(of 251))	Lab collection
TH13813	*fliK7582::bla* Δ*flgG7661*(D6-255(of 260))	Lab collection
TH13814	*fliK7582::bla* Δ*flgH7662*(D6-227(of 232))	Lab collection
TH13815	*fliK7582::bla* Δ*flgI7663*(D6-360(of 365))	Lab collection
TH13816	*fliK7582::bla* Δ*flgJ7664*(D6-311(of 316))	Lab collection
TH13817	*fliK7582::bla* Δ*flgK7665*(D6-548(of 553))	Lab collection
TH13818	*fliK7582::bla* Δ*flgL7666(D6-312(of 317))*	Lab collection
TH27867	*fliK7582::bla* Δ*flhE7404*	This study
TH27868	*fliK7582::bla* Δ*flgA7656* Δ*flhE7404*	This study
TH27869	*fliK7582::bla* Δ*flgB7657* Δ*flhE7404*	This study
TH27870	*fliK7582::bla* Δ*flgC7658* Δ*flhE7404*	This study
TH27871	*fliK7582::bla* Δ*flgD6540* Δ*flhE7404*	This study
TH27872	*fliK7582::bla* Δ*flgE7659* Δ*flhE7404*	This study
TH27873	*fliK7582::bla* Δ*flhE7404*	This study
TH27874	*fliK7582::bla* Δ*flgG7661* Δ*flhE7404*	This study
TH27875	*fliK7582::bla* Δ*flgH7662* Δ*flhE7404*	This study
TH27876	*fliK7582::bla* Δ*flgI7663* Δ*flhE7404*	This study
TH27877	*fliK7582::bla* Δ*flgJ7664* Δ*flhE7404*	This study
TH27878	*fliK7582::bla* Δ*flgK7665* Δ*flhE7404*	This study
TH27879	*fliK7582::bla* Δ*flgL7666* Δ*flhE7404*	This study

Table S3: Plasmids used in this study

Name	Genotype and usage	Source or Reference
pKD46	Lambda Red	[3]
pWRG730	Lambda Red	[4]
pCP20	FRT flipping	[5]
pKD4	FKF cassette insertion	[3]
pEM8731	pKH70-P_{rpsM}-*mneongreen*<br>Visualisation cells. Constitutive promoter.	[6]
pEM8313	pKH70-P_{rpsM}-*mcerulean*<br>Visualisation cells. Constitutive promoter	[6]
pEM8913	pKH70-P_{rpsM}-*flhE*<br>Complementation *flhE.* Constitutive promoter	This study
pXY027	pCA24N-*ftsZ-gfp* (C-terminal, non-functional)	Addgene Plasmid #98915 [2]
pEM12582	pCA24N- *mreB-mneongreen-mreB* (Between aa G228-D229, SAGASA linker)<br>Visualisation of MreB by confocal microscopy	This study
pTrc99AFF4	Empty vector, IPTG inducible promoter	[7]
pTrc99A FF4-*fliC*$^{+}$	Expression of *fliC* full length (CDS aa 1 – aa 495)	Lab collection
pEM14674 / pTrc99A FF4-*fliC* (Δ aa 451-495)	Expression of *fliC* C-terminal truncated (CDS aa 1 – aa 450)	This study
pTrc99AFF4-*flgE*$^{+}$	Expression of hook subunits *flgE*	Lab collection
pTrc99AFF4-*flgJ*$^{+}$	Expression of rod cap *flgJ*	Lab collection
pTrc99AFF4-*flgG*$^{+}$	Expression of distal rod subunit *flgG*	Lab collection
pTrc99AFF4-*fliS*$^{+}$	Expression of flagellin chaperone *fliS*	Lab collection
pEM12581	pCA24N- *mneongreen-minD* (N-terminal, SAGASA linker)<br>Construction of MinD-mNeonGreen	This study

Table S4: Primers used in this study

**Number**	**Name**	**Sequence (5' -> 3')**	**Usage**
3011	Δ*flhE*-FCF-fw	gaccattggaggaaaataatgcgtaaatggctggcgttGTGTAGGCTGGAGCTGCTTC	Deletion
3012	Δ*flhE*-FCF-rv	gggcggttagcggtagttcacaatcacctgattgctccgCATATGAATATCCTCCTTAG	Deletion
3028	5'-*flhE*-seq-fw	GGTATTACTGGTTAACCATGCG	Sequencing
3029	3'-*flhE*-seq-rv	ACAGTAAAAGCTAAGCTGGG	Sequencing
3876	5'-*flhE*-Cter-fw	ATGCGTAAATGGCTGGCGTT	Sequencing
3877	3'-*flhE*-Nter-rev	TTAGCGGTAGTTCACAATCACC	Sequencing
3407	5'-EcoRI-*flhE*-fw	ggacagaattcataaggaggaaaaacatATGCGTAAATGGCTGGCGTT	Cloning
3408	3'-NotI-*flhE*-rev	gtactgcggccgcTGCAGTTTTACCGCCGACGC	Cloning
4052	5'-*KanSceI-flhE*-del-Nter-fw	gtcatatccgcatgacggcgaccattggaggaaaataatgAGGGTTTTCCCAGTCACGAC	Deletion
4053	3'-*KanSceI-flhE*-del-Nter-rev	ttacacccataccgctatcctgccacgcgccttcgcctgcTGCTTCCGGCTCGTATGTTG	Deletion
4684	5'-*flhBA*-FKF-fw	gtggcagaagagagcgacgacgacaaaacagaagcccccaGTGTAGGCTGGAGCTGCTTC	Deletion
4685	3'-*flhB*-FKF-rev	tccagcgcttctggcaccggaaggttctcaggttgtggagCATATGAATATCCTCCTTAG	Deletion
4686	5'-*flhA*-FKF-fw	ggtcgcgatgctgcgcctgcccagcaacctgaaatcgacgGTGTAGGCTGGAGCTGCTTC	Deletion
4687	3'-*flhBA*-FKF-rev	atatgacggttatcggaaagctcaaggttcgacaacaccaCATATGAATATCCTCCTTAG	Deletion
4688	5'-Δ*fliPQR*-FRT-fw	ggagatcctgatgcgccgtttgttattcctttctctggcgGTGTAGGCTGGAGCTGCTTC	Deletion
4689	3'-Δ*fliP*_aa10-241-FRT-rev	gattcaggagtcattttgcgcctctaactgtaaaagctttCATATGAATATCCTCCTTAG	Deletion
4690	3'-Δ*fliPQR*-FRT-rev	ttatgggttattatttatcggcatctcgctaacaatatcaCATATGAATATCCTCCTTAG	Deletion
5400	5'-G228-*mreB*-overlapping	ATGTTGAAAAAATTTCGTGG	MreB-mNG Gibson Assembly
5401	3'-G228-*mreB*-overlapping	tgctgatgcgcctgcacttgaGCCCGGATAAGCGGAACCGA	MreB-mNG Gibson Assembly
5402	5'-mNG-overlapping	tcaagtgcaggcgcatcagcaATGGTATCGAAGGGCGAGGA	MreB-mNG Gibson Assembly
5404	3'-mNG-Venus-overlapping	ggccgaagcacctgcggaaccTTTATACAGTTCATCCATGC	MreB-mNG Gibson Assembly
5405	5'-D229-*mreB*-overlapping	ggttccgcaggtgcttcggccGACGAAGTCCGCGAGATCGA	MreB-mNG Gibson Assembly
5406	3'-D229-*mreB*-overlapping	CTACTCTTCGCTGAACAGGT	MreB-mNG Gibson Assembly
5407	5'-pXY027-*mreB*-fw-GA	acaggtcattggaaaacatgccacgaaatttttcaacatACTAGTAGTTAATTTCTCCT	MreB-mNG Gibson Assembly
5408	3'-pXY027-*mreB*-rev-GA	atgatcgatatgcacggcggcgacctgttcagcgaagagtagGCGGCCGCTAAGGGTCGA	MreB-mNG Gibson Assembly
5409	5'-mNG-*minD*-GA-fw	tttcacacagaattcattaaagaggagaaattaactactagtATGGTATCGAAGGGCGAG	MinD-mNG Gibson Assembly
5410	3'-mNG-*minD*-GA-rev	acaacaataatgcgtgccattgctgatgcgcctgcactTTTATACAGTTCATCCAT	MinD-mNG Gibson Assembly
5411	5'-*minD*-GA-fw	agtgcaggcgcatcagcaATGGCACGCATTATTGTTGT	MinD-mNG Gibson Assembly
5412	3'-*minD*-GA-rev	cagctaattaagcttggctgcaggtcgacccttagcggccgcTTATCCTCCGAACAGGCG	MinD-mNG Gibson Assembly
5413	5'-pXY027-*minD*-GA-fw	gcggccgctaagggtcgacc	MinD-mNG Gibson Assembly
5414	3'-pXY027-*minD*-GA-rev	actagtagttaatttctcct	MinD-mNG Gibson Assembly
2522	*fliC*-G426-*tetRA*-FW	gctgctttggcacaggttgacacgttacgttctgacctggTTAAGACCCACTTTCACATT	*fliC* point mutant
2523	*fliC*-A449-*tetRA*-RV	aaacttcggtcgcgtagtcggaatcttcgatacggctacgCTAAGCACTTGTCTCCTG	*fliC* point mutant
2524	*fliC*-G426A-FW	gctgctttggcacaggttgacacgttacgttctgacctggCtgcggtacagaaccgtttc	*fliC* point mutant
3751	5'-*fliC*-A427V-fw	gctgctttggcacaggttgacacgttacgttctgacctgggtgTggtacagaaccgtttc	*fliC* point mutant
2526	*fliC*-1484-RV	cgcagtaaagagaggacgtttt	*fliC* point mutant
3752	3'-*fliC*-A449T-rev	aacttcggtcgcgtagtcggaatcttcgatacggctacgCGTagaagtcaggttgtttac	*fliC* point mutant
2525	*fliC*-A449V-RV	aacttcggtcgcgtagtcggaatcttcgatacggctacggAcagaagtcaggttgtttac	*fliC* point mutant
3753	3'-*fliC*-Q472L-rev	cctggttcgcctgcgccagaacggaggtaccggcctgcagCAGAATCTGCGCGCGAGACA	*fliC* point mutant
2527	*fliC*-1147-FW	gcaagtaaagccgaaggtca	*fliC* point mutant
202	5'-*fliC*end-*tetR*_fw	ggcgaaccaggttccgcaaaacgtcctctctttactgcgtTTAAGACCCACTTTCACATT	*fliC* transcriptional fusion
203	3'-*fliC*end-3'UTR-*tetA*_rv	tgccttgattgtgtaccacgtgtcggtgaatcaatcgccggaCTAAGCACTTGTCTCCTG	*fliC* transcriptional fusion
3395	5'-mNeonGreen-*fliC*-trans	gaaccaggttccgcaaaacgtcctctctttactgcgttaaGAATTCATAAGGAGGAAAAA	*fliC* transcriptional fusion
3396	3' -mNeonGreen-*fliC*-trans	ccttgattgtgtaccacgtgtcggtgaatcaatcgccggaTTATCATTTATACAGTTCAT	*fliC* transcriptional fusion
619	*flhDC*-P5x_fw_52	CATAAGACACTTTTTACACACCAC	$P_{pro/syn}$-*flhDC*
3718	*3'-PproC-flhD_rv*	tgtgtagagggaaaccgttgtggtctccctgaatatATCATAcgagccttatgcatgccc	$P_{pro/syn}$-*flhDC*
3719	3'-*PproB-flhD*_rv	tgtgtagagggaaaccgttgtggtctccctgaatatATATTAcgagccttatgcatgccc	$P_{pro/syn}$-*flhDC*
3720	*3'-PproA-flhD_rv*	tgtgtagagggaaaccgttgtggtctccctgaatatAGCCTAcgagccttatgcatgccc	$P_{pro/syn}$-*flhDC*
3721	3'-*Ppro1-flhD*_rv	tgtgtagagggaaaccgttgtggtctccctgaatatAGATACcgagccttatgcatgccc	$P_{pro/syn}$-*flhDC*
3251	*5'dPflhDC(-598)-proD_ fw*	actttttacacaccacccggatgcttcatttaaatgggtgCAGCTAACACCACGTCGTCC	$P_{pro/syn}$-*flhDC*
3255	3'-RBS(*proD*)-*flhDC*_rv	ATAAATGTGTTTTAGCAACTCGGATGTATGCATTGTTCCCATctagtaCTTTCCTGTGTG	$P_{pro/syn}$-*flhDC*
2884	FliK*bla*-fw	caggccgccgcgcgcggcaatggcgcagtggatatcttcgcccacccagaaacgctggtg	FliK-Bla fusion
2885	FliK*bla*-Rev	gcgtggaaaagacgcggataatcatgctacctctggcgttaccaatgcttaatcagtgag	FliK-Bla fusion

Supplementary References

1. Shaner, N. C. *et al.* A bright monomeric green fluorescent protein derived from *Branchiostoma lanceolatum*. *Nature Methods* **10**, 407–409 (2013).
2. Buss, J. *et al.* A multi-layered protein network stabilizes the *Escherichia coli* FtsZ-ring and modulates constriction dynamics. *PLoS Genet* **11**, e1005128 (2015).
3. Datsenko, K. A. & Wanner, B. L. One-step inactivation of chromosomal genes in *Escherichia coli* K-12 using PCR products. *Proceedings of the National Academy of Sciences* **97**, 6640–6645 (2000).
4. Blank, K., Hensel, M. & Gerlach, R. G. Rapid and Highly Efficient Method for Scarless Mutagenesis within the *Salmonella enterica* Chromosome. *PLOS ONE* **6**, e15763 (2011).
5. Cherepanov, P. P. & Wackernagel, W. Gene disruption in *Escherichia coli* : TcR and KmR cassettes with the option of Flp-catalyzed excision of the antibiotic-resistance determinant. *Gene* **158**, 9–14 (1995).
6. Delgadillo-Guevara, M., Halte, M., Erhardt, M. & Popp, P. F. Fluorescent tools for the standardized work in Gram-negative bacteria. *J Biol Eng* **18**, 25 (2024).
7. Ohnishi, K., Fan, F., Schoenhals, G. J., Kihara, M. & Macnab, R. M. The FliO, FliP, FliQ, and FliR proteins of *Salmonella* Typhimurium: putative components for flagellar assembly. *J Bacteriol* **179**, 6092–6099 (1997).
