# Supplementary material for: FlhE functions as a chaperone to prevent formation of periplasmic flagella in Gram-negative bacteria
Source: Nat Commun. 2024 Jul 14;15:5921. doi: 10.1038/s41467-024-50278-0 (PMC11247099; doi:10.1038/s41467-024-50278-0)
Supplement: Supplementary file 3 — Description of Additional Supplementary Files [file 41467_2024_50278_MOESM3_ESM.pdf]

Description of Additional Supplementary Files:

Supplementary Dataset 1: The presence of FlhE domain containing proteins across representative set of bacterial species (GTDB, v.95). Only genomes with identified FlhE domain are listed in the table.For each FlhE domain containing protein its identifier, domain architecture, and length are shown. For proteins that are available in MiST 4 database neighborhood information is shown.

Supplementary Movie 1: Phase contrast time-lapse imaging showing 3 exemplary cells (PtetA-flhDC ΔflgHI ΔflhE) after 2,5 hours induction of PtetA-flhDC with AnTc. Some cells are able to rotate their cell body, either attached to the coverslip (left panel), rotating in a circular motion (middle panel) or rotating the cell body in a similar manner to bacterial cell from the Leptospira genus. Scale bar = 10 µm and time in milliseconds are indicated (43 msec /frame).
